# *Cydonia oblonga* Mill. Fruit Extract Ameliorates Lipopolysaccharide-Induced Depression-like Behaviors in Mice by Modulating Inflammation, Metabolites and Gut Microbiota

**DOI:** 10.3390/nu18142322

**Published:** 2026-07-16

**Authors:** Gusonghan Maitiniyazi, Shan Zhao, Ayinuer Abulikemu, Yibadaiti Aihemaiti, Yu-Xin Song, Shu-Fang Xia

**Affiliations:** 1 School of Nursing, Xinjiang Hetian College, Hetian 848000, China; aynur18609038818@163.com (A.A.); 19039951093@163.com (Y.A.); yuxin7621@163.com (Y.-X.S.); 2 Xinjiang Key Laboratory of Hetian Characteristic Chinese Traditional Medicine Research, Hetian 848000, China; 18197763241@163.com; 3 School of Basic Medicine, Xinjiang Hetian College, Hetian 848000, China; 4 Wuxi School of Medicine, Jiangnan University, Lihu Avenue 1800, Wuxi 214122, China

**Keywords:** depression, *Cydonia oblonga* Mill., inflammation, TLR4/MyD88/NF-κB, gut microbiota, metabolites

## Abstract

Background: *Cydonia oblonga* Mill. is rich in flavonoid compounds and shows potential for application in functional foods. This study investigated the therapeutic potential of hydroethanolic extract of *Cydonia oblonga* Mill. fruit (HECO) in alleviating lipopolysaccharide (LPS)-induced depression-like behaviors in mice. Methods: Mice were treated with HECO (600 mg/kg, p.o.) for 21 consecutive days. From day 14 to day 21, depression-like behavior was induced by LPS (2.0 mg/kg injected i.p.). Behavioral parameters and biochemical markers were then assessed. Gut microbiota composition was analyzed by 16S rRNA gene sequencing, and serum metabolites were profiled through untargeted metabolomics. Results: HECO improved LPS-induced behavioral alterations, including increased locomotor activity in the open-field test and decreased immobility time in the forced swimming test and tail suspension test, and reduced serum IL-6 levels in LPS-treated mice. Furthermore, HECO markedly upregulated the expression of occludin and ZO-1 in the hippocampus and inhibited neuroinflammation by suppressing activation of the TLR4/MyD88/NF-κB signaling pathway. HECO significantly increased the relative abundance of Deferribacterota in LPS-induced mice. A total of 262 differential metabolites were identified between the LPS and HECO groups, with the top potential biomarkers predominantly categorized into lipids, flavonoid-containing phenylpropanoids, organic acids, and peptides. Conclusions: HECO improved depression-like behavior in LPS-induced mice, potentially through modulation of gut microbiota and serum metabolites, attenuation of systemic inflammation, and inhibition of the TLR4/MyD88/NF-κB signaling pathway in the hippocampus.

## 1. Introduction

Depression is a common mental disorder characterized by prolonged low mood and loss of pleasure or interest in activities, and individuals with severe depression may experience thoughts of death or suicide [1]. According to the World Health Organization’s (WHO) 2023 estimates, depression affects approximately 280 million people worldwide [2], making it a leading cause of disability and a major contributor to the global disease burden. Currently, first-line clinical treatments for depression include antidepressant medications and psychotherapy [3]. Although antidepressants based on the monoamine hypothesis are effective, they are associated with low response rates and undesirable adverse effects [4,5]. Therefore, there is a need to develop mild complementary therapies to improve treatment efficacy and tolerability.

Despite various hypotheses, the exact pathological mechanisms underlying depression are still not fully understood. Contributing factors include environmental influences, genetics, neuroendocrine and neurotransmitter abnormalities, inflammation, metabolic dysfunction, and gut microbiome dysbiosis [6,7]. Increasing preclinical and clinical studies have highlighted that compositional and functional (e.g., metabolite) changes in gut microbiota are associated with the onset and progression of depression via the gut–brain axis [8,9]. However, gut microbiota and their metabolites may exert both protective and detrimental effects in depression, partly through modulation of neuroinflammatory pathways. Dysbiosis has been implicated in the pathogenesis of depression and has emerged as a potential therapeutic target [9]. Furthermore, inflammation is recognized a major pathogenic factor contributing to depression, and the toll-like receptor 4/myeloid differentiation factor 88/nuclear factor-κB (TLR4/MyD88/NF-κB) signaling pathway plays a central role in inflammatory responses. Studies confirm that lipopolysaccharide (LPS)-induced neuroinflammation is linked to the activation of the TLR4/MyD88/NF-κB pathway [10,11]. Specifically, LPS activates the TLR4 pattern recognition receptor on microglia, which in turn engages the MyD88 adaptor protein, leading to the nuclear translocation of NF-κB and the subsequent release of pro-inflammatory cytokines (e.g., interleukin (IL)-6, IL-1β, and tumor necrosis factor (TNF-α)), chemokines, and inducible enzymes (e.g., cyclooxygenase-2 (COX-2), and inducible nitric oxide synthase (iNOS)), which collectively contribute to neuroinflammatory responses [12]. Consequently, the TLR4/MyD88/NF-κB axis represents a promising target for depression therapies.

**Figure 1:**
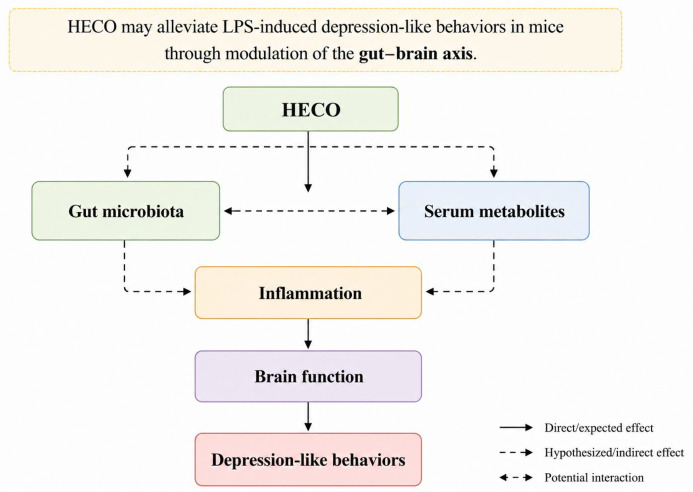
A schematic diagram of the study hypothesis. HECO administration is proposed to modulate gut microbiota composition and serum metabolites, reduce neuroinflammation, and improve depressive-like behaviors in LPS-induced mice. Dashed arrows indicate hypothesized relationships that require further experimental validation.

Dietary flavonoids, a class of plant-derived natural compounds, show potential in improving depressive symptoms by modulating the gut microbiota and their metabolites [13,14] and reducing inflammatory responses [15,16]. *Cydonia oblonga* Mill. (COM, also known as quince), a member of the Rosaceae family, is widely used for nutritional and medicinal purposes due to its high content of flavonoids [17]. COM is known for its many therapeutic effects, including antioxidant, anti-inflammatory, antimicrobial, anti-ulcerative, and anticancer actions [18]. COM polyphenol extract has been shown to inhibit the activation of NF-κB mediated by LPS, thereby reducing the secretion of IL-8 and TNF-α [19]. Hydroethanolic extract of COM fruits (HECO) has demonstrated antioxidant potential that protects dopaminergic neurons from oxidative damage [20] and also enhances hippocampal neurogenesis while preventing depressive-like behaviors rats with chronic immobilization stress [21]. However, the effects of HECO on LPS-induced depression-like behaviors remain unclear. The proposed working hypothesis of the present study is summarized in Figure 1, which illustrates the potential pathways through which HECO may exert its antidepressant effects.

Accordingly, the present study aimed to investigate the effects of HECO on LPS-induced depression-like behaviors, neuroinflammation, gut microbiota and metabolomics in mice. Special emphasis was placed on the potential involvement of the TLR4/MyD88/NF-κB pathways in the protective effects of HECO.

## 2. Materials and Methods

### 2.1. Preparation of HECO

Healthy COM fruits showing no signs of insect damage or fungal infection were collected from Kashgar, Xinjiang, China. Voucher specimens (2021−11−3) were deposited at Wuxi School of Medicine, Jiangnan University, People’s Republic of China. The preparation of HECO from COM fruits and the determination of its flavonoid components by UPLC-MS were performed according to our previously published method [22]. After washing the fruits with tap water, the seeds were removed, and the fruits were cut into thin slices and dried. The dried slices were ground into a fine powder using an electric mill. The powder was then extracted in 65% ethanol at a solid-to-solvent ratio of 1:20 (*w*/*v*) and the mixture was subjected to ultrasonic treatment at 60 °C with an ultrasonic frequency of 40 kHz for 30 min. After ultrasonication, the crude extract was filtered through filter paper (pore size 11 μm) under vacuum. The filtrate was then centrifuged at 3000 rpm for 10 min. After centrifugation, the supernatant was carefully collected, and the pellet was discarded. This centrifugation and supernatant collection step was repeated three times, and the three supernatants were pooled [23]. The pooled supernatant was then concentrated using a rotary evaporator at 50 °C under reduced pressure (150 mbar) at a rotation speed of 80 rpm until dryness. Finally, the concentrated extract was freeze-dried to yield HECO for subsequent experiments.

### 2.2. Determination of Flavonoid Components by UPLC-MS

UPLC-MS was used to analyze the flavonoid composition of HECO. Briefly, 20 mg of freeze-dried HECO powder was extracted with 600 μL methanol, sonicated for 15 min, and centrifuged at 12,000 rpm and 4 °C for 5 min. The supernatant was filtered through a 0.22 μm membrane filter. Chromatographic separation was performed on a Waters ACQUITY UPLC^®^ BEH C18 column ((Waters Corporation, Milford, MA, USA; 2.1 × 100 mm, 1.7 μm) at 40 °C. The mobile phase consisted of 0.1% formic acid in water (A) and methanol (B) at a flow rate of 0.25 mL/min, using a gradient elution program (10% to 90% B over 22 min). The injection volume was 2 μL. Mass spectrometry was performed on an AB Sciex 4000 QTRAP mass spectrometer (AB Sciex, Framingham, MA, USA) with an ESI source in negative ion mode. The ion source temperature was 500 °C, with an ion spray voltage of –4500 V; curtain gas, collision gas, nebulizer gas, and auxiliary gas were set at 30, 6, 50, and 50 psi, respectively. Data were acquired in multiple reaction monitoring (MRM) mode and processed using Analyst^®^ software (version 1.6, AB Sciex), with three replicates per sample. The main flavonoids identified in HECO were rutin, L-epicatechin, catechin, and glycitin.

### 2.3. Animals

This study was approved by the Experimental Animal Care and Use Ethics Committee of Xinjiang Medical University (IACUC-JT-20240415-20). Male C57BL/6J mice aged 6~7 weeks were obtained from SPF Biotechnology Co., Ltd. (Beijing, China) and housed in a room under controlled temperature (23 ± 2 °C) and humidity (60 ± 5%), and kept under a 12 h light/dark cycle with free access to food and water. All experiments complied with the ARRIVE guidelines and carried out in accordance with the National Research Council’s Guide for the Care and Use of Laboratory Animals. 

### 2.4. Experimental Protocol

**Figure 2:**
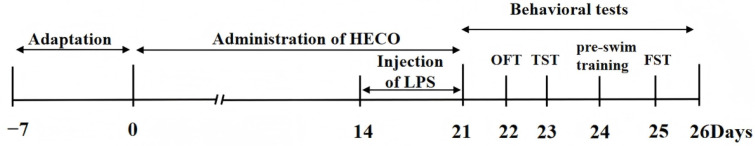
Schematic illustration of study.

A total of 30 male C57BL/6 mice were used in this study. After 1 week of acclimatization, mice were randomly divided into three groups (10 mice per group): (1) control group (Con, *n* = 10); (2) LPS- induced model group (LPS, *n* = 10); (3) LPS with HECO intervention group (HECO, *n* = 10). The first day after acclimatization was considered day 1. From day 1 to day 21, mice in the HECO group were treated with 600 mg/kg HECO by oral gavage at a volume of 10 mL/kg body weight, whereas mice in the Con and LPS groups were given the same volume of vehicle (distilled water) by oral gavage. The HECO dose of 600 mg/kg was selected according to our previous study [22], which demonstrated its efficacy in improving depression-like behaviors in mice. From day 14 to day 21, mice in the LPS and HECO groups received intraperitoneal injections of LPS (2.0 mg/kg, Abmole Bioscience Inc., Houston, TX, USA) dissolved in 0.9% NaCl solution at a volume of 10 mL/kg body weight, whereas mice in the Con group were injected with the same volume of 0.9% NaCl solution. LPS was administered once daily. Twenty-four hours after the last oral administration, mice were submitted to behavioral tests and then euthanized. A diagram of the experimental design is shown in Figure 2.

### 2.5. Behavioral Measurements

#### 2.5.1. Open-Field Test (OFT)

The OFT [24] was performed in a square box (40 cm × 40 cm × 30 cm) with no ceiling, located in a dark and quiet room. No prior training is required for this test; however, the mice were habituated to the testing room for 30 min before the test. The bottom of the arena was divided into 16 identical squares (10 cm × 10 cm), where 4 central squares were designated as the core zone. The mice were placed at the central area and allowed to explore freely for 6 min. Percent of time spent in different areas, total distance traveled, moving distance in the central areas, and average moving speed in the last 5 min were recorded as measures of anxiety-related behavior and ambulatory activity.

#### 2.5.2. Tail Suspension Test (TST)

The mice were suspended by their tails from a horizontal bar, with their heads positioned 15 cm above the ground. This test does not require prior training. Absolute lack of movement by the animal was defined as immobility, and the time spent immobile during 6 min was recorded. Following a 6 min observation window, behavioral data were recorded during the final 4 min to evaluate depression-like behavior [25]. 

#### 2.5.3. Forced Swim Test (FST)

The mice were positioned in Pyrex beakers (10 cm diameter, 25 cm height) filled with water maintained at 25 °C for 6 min [26]. Twenty-four hours before the formal test, all mice received a 15 min pre-swim training session (no data recorded) to reduce acute stress and ensure stable baseline immobility. The computation of motionlessness was carried out during the final 4 min. Periods of inactivity were recorded when the mice became thoroughly still or exhibited only minimal movements requisite for keeping their heads above the water’s surface.

### 2.6. Sample Collection

After behavioral tests, mice were fasted overnight and then anesthetized with 1% sodium pentobarbital (10 mg/mL) administered intraperitoneally at a dose of 50 mg/kg body weight (5 mL/kg). Blood samples were collected by enucleation. The blood was allowed to clot at room temperature for 30 min and then centrifuged at 3000 rpm for 10 min at 4 °C to obtain serum. Subsequently, the mice were euthanized by decapitation, and the organs (whole brain, kidney, spleen, liver, testis) were isolated and weighed, and the hippocampus and colonic contents were harvested and stored at −80 °C for further analysis.

### 2.7. Hematoxylin–Eosin (H&E) and Nissl Staining

After fixation in 4% paraformaldehyde (PFA), whole brains were subjected to graded ethanol dehydration and xylene rinsing. Subsequently, the samples were embedded in paraffin and sectioned into 5 μm slices and cover-slipped using a standard mounting medium for hematoxylin and eosin (H&E) staining. Nissl staining was used to detect histological and distributional changes in hippocampal neuronal morphology using the Nissl staining kit (Solarbio, Beijing, China). Finally, tissue sections were imaged using a Pannoramic MIDI Slide scanner (3D Histech, Budapest, Hungary).

### 2.8. Immunofluorescence (IF)

The fixed brain tissues were embedded, and serial coronal sections (50 μm thick) were obtained using a microtome. After washing three times with PBS, the sections were incubated overnight (approximately 16 h) at 4 °C with primary antibody working solution (1:200 dilution in 3% BSA and 10% normal donkey serum). The primary antibodies used in this experiment was rabbit anti-parvalbumin (PV) (1:200, ab181086, Abcam, Cambridge, UK). After primary antibody incubation, the sections were thoroughly rinsed and then incubated with HRP-conjugated goat anti-rabbit IgG secondary antibody (1:500, Servicebio, Wuhan, China) at room temperature for 2 h. After secondary antibody retrieval, the brain sections were thoroughly washed with PBS, then incubated with 4′,6-Diamidino-2-phenylindole (DAPI) for 10 min. Following extensive rinsing, the sections were mounted with an anti-fade mounting medium. After drying in the dark, the samples were imaged using fluorescence microscopy. PV-positive cells were counted in three randomly selected fields per section from three sections per mouse, and the analyst was blinded to the group allocation. The region of interest (ROI) was defined as the entire hippocampal regions (CA1, CA3, and DG).

### 2.9. Enzyme-Linked Immunosorbent Assay (ELISA)

The levels of TNF-α, IL-6, IL-1β, COX-2, and iNOS in serum were measured using corresponding ELISA kits (Meimian, Yancheng, Jiangsu, China) according to the manufacturer’s instructions.

### 2.10. Western Blot Analysis

The hippocampal tissues were homogenized in precooled enhanced RIPA lysis buffer (Beyotime, Shanghai, China) containing protease inhibitor cocktail (1:100, *v*/*v*; Beyotime, Shanghai, China), followed by lysis on ice for 15 min. Subsequently, the homogenate was centrifuged at 12,000 rpm for 20 min at 4 °C to obtain the supernatant. Ten microliters of protein per sample was resolved on a precast SDS-PAGE gel using Tris-glycine SDS running buffer at 120 V for 90 min. The separated proteins were then transferred onto polyvinylidene fluoride (PVDF) membrane in transfer buffer (25 mM Tris, 192 mM glycine, 20% methanol) at 100 V for 60 min on ice. After blocking with 5% skim milk powder in TBST (Tris-buffered saline with 0.1% Tween-20) for 1 h at room temperature, the membrane was washed three times with TBST (5 min each). The membrane was then incubated with different primary antibodies diluted in TBST: rabbit anti-TLR4 (PAB33926, Bio-Swamp, Wuhan, China), rabbit anti-MyD88 (PAB47936, Bio-Swamp, Wuhan, China), rabbit anti-NF-κB p65 (RMAB50740, Bio-Swamp, Wuhan, China), rabbit anti-ZO-1 (PAB36669, Bio-Swamp, Wuhan, China), rabbit anti-Occludin (PAB46094, Bio-Swamp, Wuhan, China), and mouse anti-β-actin (Proteintech, Wuhan, China) overnight at 4 °C. After washing three times with TBST (5 min each), the membrane was incubated with peroxidase-conjugated secondary antibodies (goat anti-mouse IgG or goat anti-rabbit IgG, diluted 1:5000 in TBST) for 1 h at room temperature. Following three additional washes with TBST (5 min each), detection was conducted with enhanced chemiluminescence reagents (Immobilon Western Detection Reagents, Millipore, Bedford, MA, USA) and exposure to film.

### 2.11. DNA Extraction and 16S rRNA Sequencing

Total DNA was extracted from feces (*n* = 6 randomly selected from each group) using the microbial E.Z.N.A.^®^ soil DNA Kit (Omega Bio-Tek, Norcross, GA, USA). Gel electrophoresis was employed to assess the purity and concentration of the extracted DNA. Specifically, 5 μL of DNA sample was loaded onto a 1% agarose gel containing 0.5 μg/mL ethidium bromide and electrophoresed in 1× TAE buffer at 120 V for 30 min. A DNA ladder (DL2000) was used as a size marker. The sample was diluted with RNase-Free Water and then utilized as a template for subsequent amplification reactions. The V3-V4 region of the bacterial 16S rRNA gene was amplified by PCR with the primers 338F (5′-ACTCCTACGGGAGGCAGCAG-3′) and 806R (5′-GGACTACHVGGGTWTCTAAT-3′). The PCR conditions were: initial denaturation at 94 °C for 3 min, followed by 30 cycles of 94 °C for 30 s, 55 °C for 30 s, and 72 °C for 30 s, with a final extension at 72 °C for 5 min. Post-purification by agarose gel electrophoresis, the PCR products were employed to construct a library and subsequently performed on an Illumina MiSeq PE300 platform (Illumina, San Diego, CA, USA) at Majorbio Bio-Pharm Technology Co., Ltd. (Shanghai, China). The reads were filtered and assembled into Tags by FLASH (version 1.2.7), the tags were then binned into operational taxonomic units (OTUs) based on 97% identification by UPARSE (version 7.1). Data were analyzed on a free online platform, Majorbio Cloud Platform (https://www.majorbio.com, accessed on 8 July 2025).

### 2.12. Non-Targeted Metabolomics

Metabolites were extracted from serum samples (6 samples per group) by adding three volumes of methanol, followed by vortexing for 30 s, and centrifuging at 12,000 rpm for 10 min at 4 °C. The supernatant was collected for LC-MS analysis. LC-MS analysis was performed on a UHPLC-Orbitrap Exploris 240 system (Thermo Fisher Scientific, Waltham, MA, USA) using an HSS T3 column, with a gradient elution of 0.1% formic acid in water/acetonitrile and 0.1% formic acid in acetonitrile/isopropanol/water at 0.40 mL/min. The resulting LC-MS raw data were processed using the metabolomics software program Progenesis QI version 3.1 (Waters Corporation, Milford, CT, USA), the raw data were preprocessed including noise filtering, baseline correction, peak calibration, peak detection, and deconvolution to obtain the final data matrix. The SIMCA (version 13.0) method was utilized to develop the model, after which model detection, principal component analysis, and correlation analysis were conducted. Meanwhile, their MS and MSMS mass spectral information as searched against and matched with that in two public metabolic databases, HMDB (http://www.hmdb.ca/, accessed on 22 May 2024) and Metlin (https://metlin.scripps.edu/, accessed on 22 May 2024), to obtain each metabolite’s information. Metabolites with VIP > 1 and *p* < 0.05 were considered significant.

### 2.13. Statistical Analysis

Statistical analysis was performed using SPSS 27.0 (SPSS, Inc., IBM Corp., Armonk, NY, USA) and GraphPad Prism 8.0 (GraphPad Software, San Diego, CA, USA). Data were presented as either median (25th, 75th percentile) or mean ± SD. One-way analysis of variance (ANOVA) followed by Bonferroni post hoc test was used for normally distributed data, whereas the Kruskal–Wallis test was applied for non-normally distributed data. A two-way ANOVA with time as repeated measure was used for the analysis of body weight. To explore potential associations among behavioral outcomes, inflammatory markers, gut microbial taxa, and representative metabolites, Spearman’s correlation analysis was performed. *p* < 0.05 was considered significant.

## 3. Results

### 3.1. Effects of HECO on Body and Organ Weight in LPS-Induced Mice

**Figure 3:**
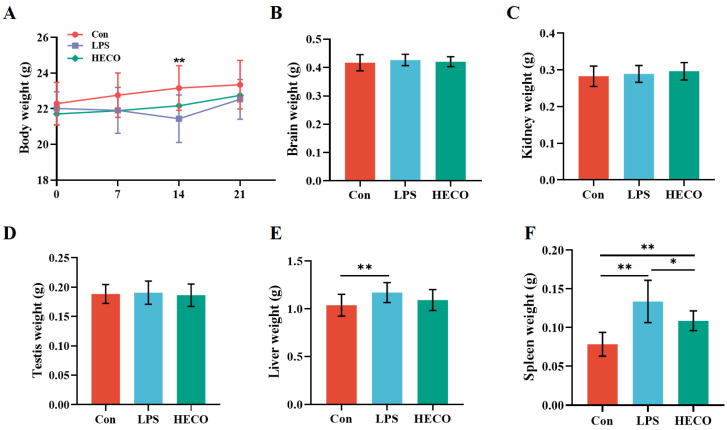
Effects of HECO on body and organs weight in LPS-induced mice. Body weight (**A**), brain weight (**B**), kidney weight (**C**), testis weight (**D**), liver weight (**E**), and spleen weight (**F**). Data are shown as mean ± SD (*n* = 10 per group). Con, control; LPS, lipopolysaccharide; HECO, LPS with hydroethanolic extract of *Cydonia oblonga* Mill. fruit intervention. * *p* < 0.05; ** *p* < 0.01.

No significant differences in body weight were observed among the Con, LPS, and HECO groups over the experimental period (*p* > 0.05), although a transient reduction was noted in the LPS group on day 14 (*p* < 0.05, Figure 3A). Similarly, no significant changes were detected in brain, kidney, or testis weights among the three groups (*p* > 0.05, Figure 3B–D). In contrast, liver weight was significantly increased in the LPS group compared with the Con group (*p* < 0.05), while HECO treatment partially reversed this effect (*p* > 0.05, Figure 3E). In addition, spleen weight was markedly elevated in the LPS group, and this increase was significantly attenuated by HECO administration (*p* < 0.05, Figure 3F).

### 3.2. Effects of HECO on Depression-like Behaviors in LPS-Induced Mice

**Figure 4:**
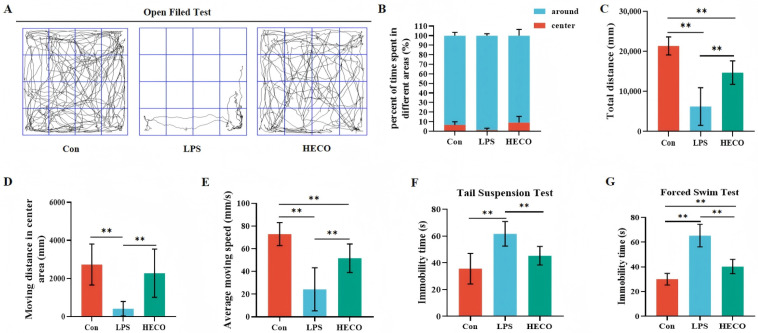
The effects of HECO on depression-like behaviors in LPS-induced mice. (**A**) The movement trajectory in the open-field test. The percent of time spent in different area (**B**), total distance (**C**), the moving distance in the center areas (**D**), and the average moving speed (**E**) were evaluated in the open-field test, respectively. (**F**) Immobility time in the tail suspension test. (**G**) Immobility time in the forced swim test. Data are shown as the mean ± SD (*n* = 10 per group). Con, control; LPS, lipopolysaccharide; HECO, LPS with hydroethanolic extract of *Cydonia oblonga* Mill. fruit intervention. ** *p* < 0.01.

Behavioral performance in the OFT was evaluated (Figure 4A–E). Compared with the Con group, the total distance traveled, the moving distance in the central area, and the average moving speed were significantly decreased in the LPS group, and these parameters were remarkably restored by HECO interventions (*p* < 0.01). Since OFT primarily reflects locomotor activity and anxiety-related behavior, these results suggest that HECO alleviated LPS-induced motor deficits and anxiety-like behavior. In the TST and FST, the LPS group showed significantly longer immobility time compared with the Con group, which was reversed by HECO treatment (Figure 4F,G; *p* < 0.01). Taken together, HECO exhibited antidepressant-like effects in LPS-stimulated mice, as evidenced mainly by improvements in behavioral despair paradigms (TST and FST), along with partial normalization of exploratory behavior in the OFT.

### 3.3. Effects of HECO on Inflammation in LPS-Induced Mice

**Figure 5:**
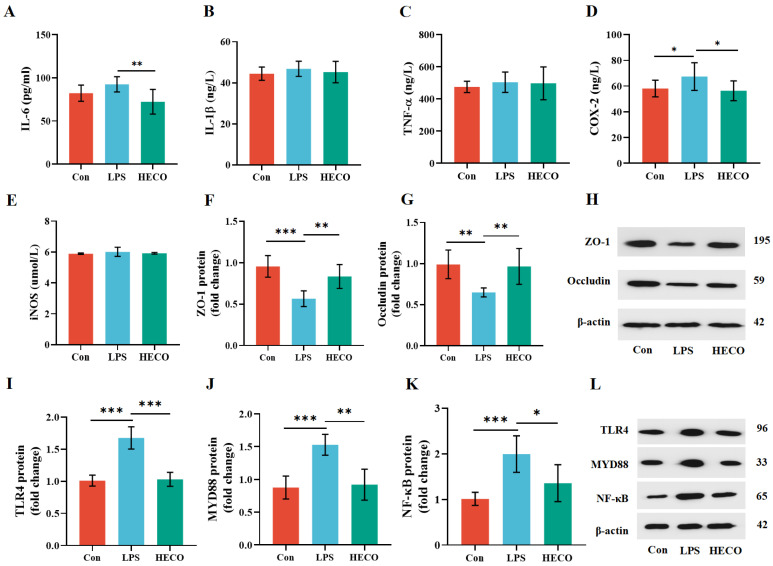
The effects of HECO on the inflammation in LPS-induced mice. Activities of IL-6 (**A**), IL-1β (**B**), and TNF-α (**C**), COX-2 (**D**), and iNOS (**E**). Relative expression of ZO-1 (**F**) and Occludin (**G**) protein in the hippocampus. (**H**) Representative Western blots of ZO-1 and Occludin in the hippocampus. Relative expression of TLR4 (**I**), MyD88 (**J**), and NF-κB (**K**) protein in the hippocampus. (**L**) Representative Western blots of TLR4, MyD88, and NF-κB in the hippocampus. ELISA data are presented as mean ± SD (*n* = 10 per group), and western blot data are presented as the mean ± SD (*n* = 6 per group). Con, control; LPS, lipopolysaccharide; HECO, LPS with hydroethanolic extract of *Cydonia oblonga* Mill. fruit intervention; ZO-1, zonula occludens-1; TLR4, Toll-like receptor 4; MyD88, myeloid differentiation primary response protein 88; NF-κB, nuclear factor kappa-light-chain-enhancer of activated B cells. * *p* < 0.05; ** *p* < 0.01; *** *p* < 0.001.

As shown in Figure 5A–C, HECO intervention significantly decreased the serum level of IL-6 in LPS group (*p* < 0.01), whereas no significant differences were observed in IL-1β and TNF-α among the three groups. COX-2 was significantly increased in the LPS group compared with the Con group, which could be remarkably decreased by HECO interventions (*p* < 0.05, Figure 5D). No significant difference was observed in iNOS expression among the three groups (Figure 5E). These findings suggest that HECO suppresses the inflammatory process by regulating the release of inflammatory mediators and the activity of related enzymes.

It is well established that tight junction proteins, including occludin and ZO-1, are downregulated in inflamed tissues, a phenomenon that facilitates the paracellular translocation of inflammatory mediators [27]. We measured the expression of ZO-1 and occludin in the hippocampus by Western blot, and the results showed that HECO significantly restored the expression of occludin and ZO-1 which were disrupted by LPS (*p* < 0.05, Figure 5F–H). Additionally, LPS induction significantly increased the expression levels of TLR4, MyD88 and NF-κB (*p* < 0.05), while HECO reversed these changes (*p* < 0.05, Figure 5I–L). Taken together, these findings suggest that HECO suppresses the inflammatory process by regulating the TLR4 signaling pathway and tight junction protein expression.

### 3.4. Effects of HECO on Hippocampal Morphology and Interneuron Number in LPS-Induced Mice

**Figure 6:**
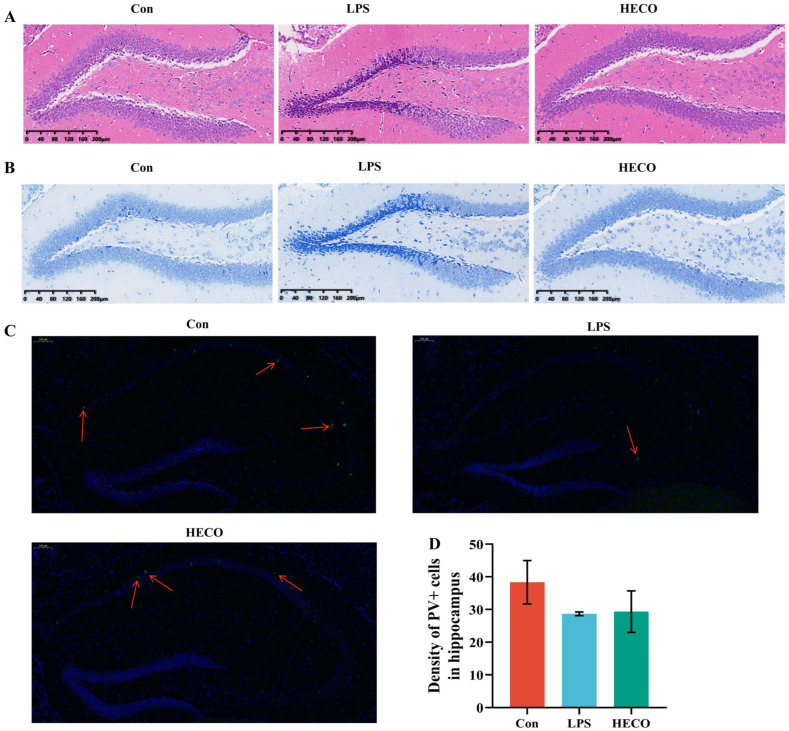
Representative photomicrographs of Hematoxylin–Eosin (H&E) staining (**A**) and Nissl staining (**B**) in the hippocampal dentate gyrus (DG) subregion. Magnification, 400×. Scale bar, 200 μm. (**C**,**D**) Immunofluorescence staining of parvalbumin (PV)-positive neurons (green) in the hippocampus with DAPI nuclear counterstaining (blue). Red arrows point to typical PV-positive neurons. Scale bar = 100 μm. Histological and immunofluorescence analyses were performed using hippocampal tissues (*n* = 3 per group). Con, control; LPS, lipopolysaccharide; HECO, LPS with hydroethanolic extract of *Cydonia oblonga* Mill.

H&E staining revealed that the pyramidal cell layer and granular cell layer in the dentate gyrus (DG) subregion were well organized and clearly layered in the Con group. In contrast, the LPS group showed slightly widened intercellular spaces within the pyramidal cell layer, irregular arrangement, and a marked reduction in the number of neurons, along with nuclear pyknosis. These pathological changes were alleviated by HECO intervention (Figure 6A). Similarly, Nissl staining showed significant neuronal loss, sparse arrangement, and reduced Nissl bodies in the DG subregion of the LPS group, which were also improved by HECO treatment (Figure 6B). Immunofluorescence staining for parvalbumin (PV) in the hippocampal region showed that PV-positive cells (green) were observed in all three groups, but no significant difference was detected among the Con, LPS, and HECO groups (Figure 6C,D).

### 3.5. Effects of HECO on Gut Microbiota Structure and Composition in LPS-Induced Mice

**Figure 7:**
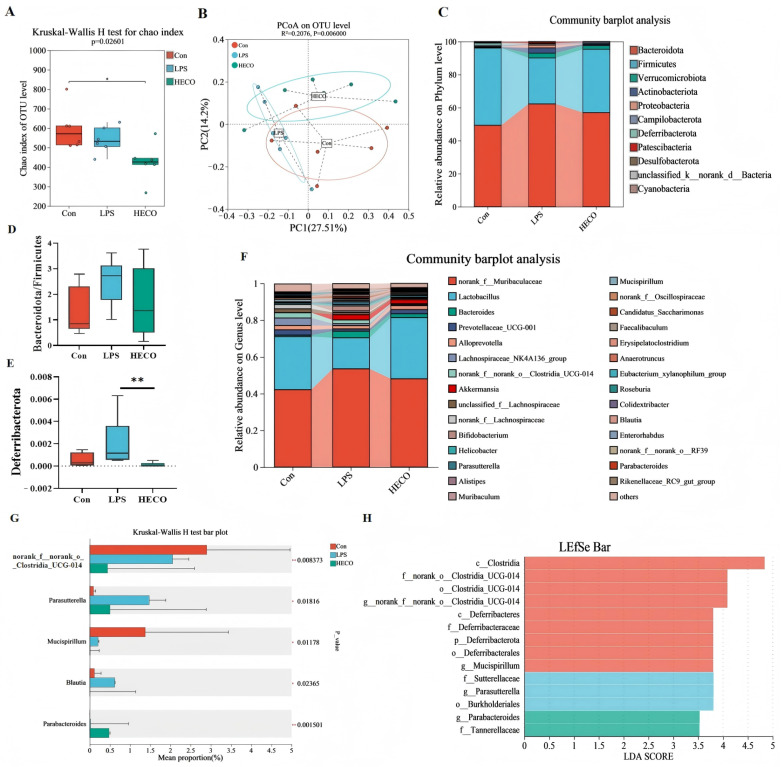
The effects of HECO on gut microbiota structure and composition in LPS-induced mice. (**A**) The Chao index was used to measure the richness of OTU. (**B**) Principal coordinate analysis (PCoA). (**C**) Relative abundance at the phylum level. (**D**) The ratio of Firmicutes/Bacteroidota. (**E**) Relative abundance of Deferribacterota. (**F**) Relative abundance at the genus level. (**G**) Five dominant bacterial genus level with significant difference. (**H**) A distribution histogram based on LDA. The sequencing data were acquired via LEfSe analysis at the phylum, class, order, family and genus levels (LDA score > 3.5). Con, control; LPS, lipopolysaccharide; HECO, LPS with hydroethanolic extract of *Cydonia oblonga* Mill. fruit intervention. *n* = 6 per group. * *p* < 0.05; ** *p* < 0.01.

To investigate the effect of HECO on the gut microbiota, 16S rRNA gene sequencing was performed. Compared with the Con group, the Chao index was significantly lower in the HECO group (*p* < 0.05, Figure 7A). PCoA illustrated that the distribution of gut microbiota among the three groups showed a tendency to separate (Figure 7B). At the phylum level, Firmicutes and Bacteroidetes were the two most dominant phyla among the three groups (Figure 7C). No significant difference was observed in the Bacteroidota/Firmicutes ratio (B/F ratio) among the three groups (Figure 7D). HECO significantly increased the relative abundance of Deferribacterota compared with the LPS group (*p* < 0.05, Figure 7E). At the genus level, *g__norank_f__Muribaculaceae* and *g__Lactobacillus* were the two most dominant genera among the three groups (Figure 7F). Several genera, including *norank_f__norank_o__ Clostridia_UCG-014*, *Parasutterella*, *Mucispirillum*, *Blautia and Parabacteroides,* showed significant difference among the three groups (Figure 7G).

LEfSe analysis (LDA score > 3.5) identified 14 differentially enriched taxa among the three groups (Figure 7H). In the HECO group, the enriched taxa included f__Tannerellaceae and *g__Parabacteroides*. In the LPS group, the enriched taxa included *g__Parasutterella*, o__Burkholderiales, and f__Sutterellaceae. In the Con group, multiple differentially enriched taxa were observed across different taxonomic levels, including c__Clostridia, f__Deferribacteraceae, *g__Mucispirillum*, p__Deferribacterota, o__Deferribacterales, f__norank_o__Clostridia_UCG-014, o__Clostridia_UCG-014, *g__norank_f__norank_o__Clostridia_UCG-014* and c__Deferribacteres.

### 3.6. Effects of HECO on Serum Metabolomics in LPS-Induced Mice

**Figure 8:**
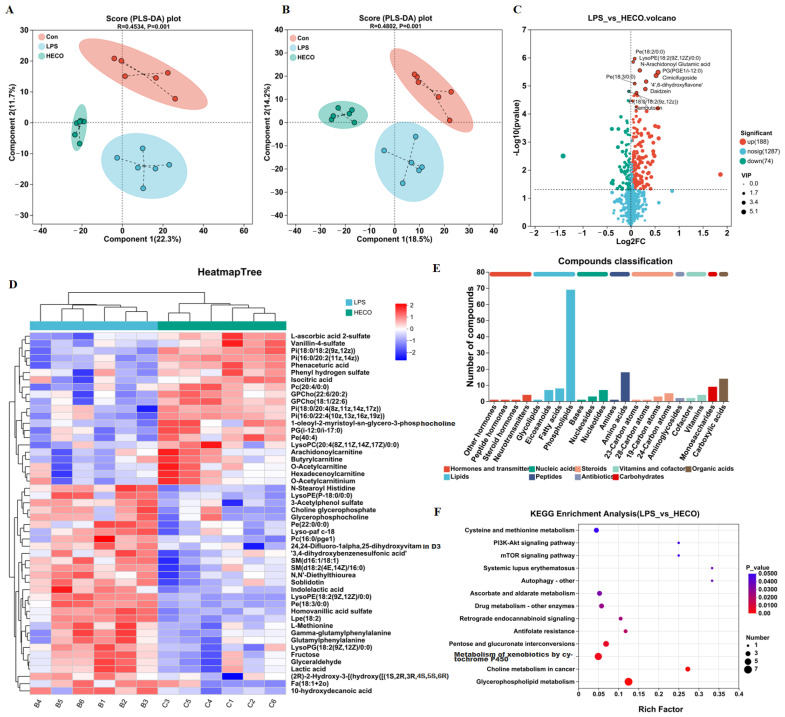
The effects of HECO on the serum metabolomic profiles in LPS-induced mice. PLS-DA score plot in the positive ion mode (**A**) and negative ion mode (**B**). (**C**) A volcano plot showing differential metabolites between the LPS and HECO groups. Metabolites are colored by significance: red (upregulated, *n* = 188), green (downregulated, *n* = 74), and blue (non-significant, *n* = 1287). (**D**) Heat map of top 50 differential metabolites between the LPS and HECO groups. (**E**) KEGG-based metabolite classification. (**F**) KEGG pathway enrichment analysis of differential metabolites between the LPS and HECO groups. Con, control; LPS, lipopolysaccharide; HECO, LPS with hydroethanolic extract of Cydonia oblonga Mill. fruit intervention. *n* = 6 per group.

Serum metabolomic profiling was performed to investigate the effects of HECO on LPS-induced metabolic alterations. A total of 262 differential metabolites were identified between the HECO and LPS groups. PLS-DA score plots showed a clear tendency of separation among the Con, LPS, and HECO groups in both positive and negative ion modes (Figure 8A,B), indicating distinct metabolic profiles among groups. Volcano plot analysis showed that 74 metabolites were significantly upregulated and 188 metabolites were significantly downregulated in the HECO group compared with the LPS group (Figure 8C). To further identify key metabolites, top 50 differential metabolites with VIP > 1.0 and *p* < 0.05 between the LPS and HECO groups were selected by OPLS-DA and visualized using a heatmap (Figure 8D). Among these, 21 metabolites were significantly upregulated and 29 metabolites were significantly decreased in the HECO group compared with the LPS group. These differential metabolites were mainly classified into lipids and lipid-like molecules (steroids and steroid derivatives, glycerophospholipids), phenylpropanoids and polyketides (flavonoids, isoflavonoids), organic acids and derivatives (organic sulfuric acids and derivatives, carboxylic acids and derivatives), benzenoids (fluorenes, benzene and substituted derivatives), organoheterocyclic compounds (naphthopyrans, benzothiazoles, isoindoles and derivatives, benzofurans) and cytochalasans.

Notably, the metabolite composition was predominantly focused on lipids (phospholipids, fatty acids, eicosanoids) and peptides (amino acids) (Figure 8E), which are associated with lipogenesis. KEGG pathway enrichment analysis of the 262 differential metabolites indicated that they were mainly involved in glycerophospholipid metabolism, PI3K-Akt signaling pathway, mTOR signaling pathway, and retrograde endocannabinoid signaling (Figure 8F). Collectively, these results indicate that HECO intervention improved LPS-induced metabolic disturbances in serum.

### 3.7. Correlation Analysis Among Behavioral, Inflammatory, Microbial, and Metabolic Parameters

**Figure 9:**
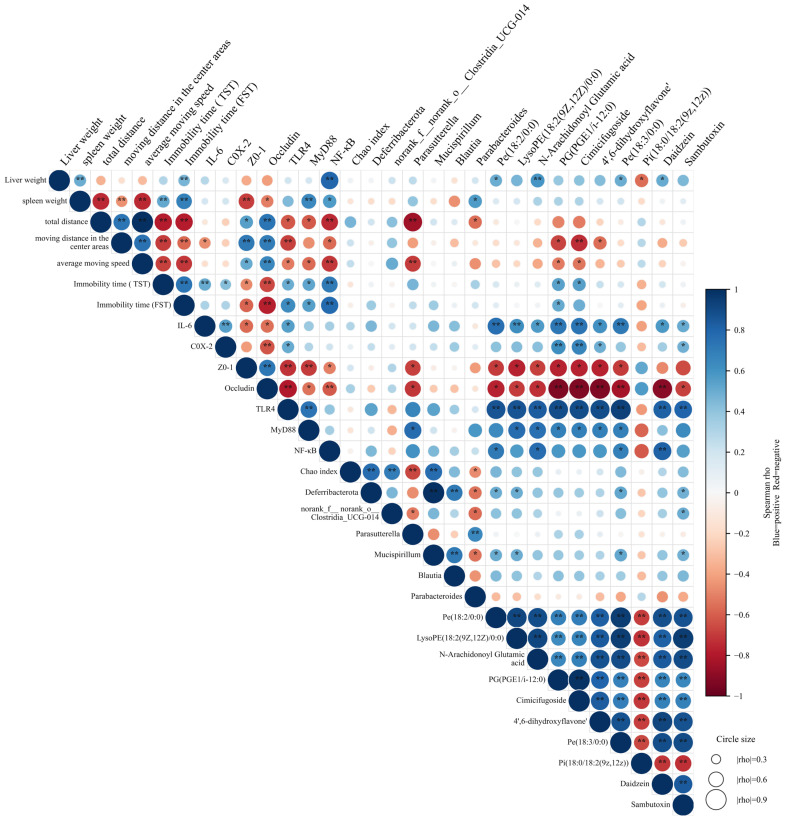
Spearman’s correlation heatmap among behavioral, inflammatory, microbial, and metabolic parameters. The color scale represents Spearman’s correlation coefficient (r): red indicates positive correlation, blue indicates negative correlation. * *p* < 0.05, ** *p* < 0.01. TST, tail suspension test; FST, forced swim test; OFT, open-field test.

As shown in Figure 9, immobility time in the TST was significantly positively correlated with IL-6, COX-2, TLR4, MyD88, and NF-κB expression, as well as specific metabolites such as PG(PGE1/i-12:0) and Cimicifugoside, while showing negative correlations with occludin and ZO-1 levels. Similarly, immobility time in the FST was positively correlated with TLR4, MyD88, and NF-κB expression, and PG(PGE1/i-12:0), and negatively correlated with occludin and ZO-1 levels. Overall, the correlation analysis suggests coordinated alterations among behavioral phenotypes, inflammatory responses, gut microbiota-related metabolites, and metabolic pathways following HECO treatment. However, these associations do not imply causality.

## 4. Discussion

Emerging research highlights the significant impact of gut microbiota, metabolites imbalances, and neuroinflammation on the onset of depression [13,28,29]. In the present study, HECO, enriched with flavonoids such as rutin, L-epicatechin, and catechin, significantly improved depressive-like behaviors in LPS-induced mice. These effects were associated with modulation of gut microbiota composition, regulation of serum metabolites, and suppression of the TLR4/MyD88/NF-κB-mediated inflammatory pathway. Collectively, these findings suggest that HECO exerts antidepressant-like effects, potentially through the attenuation of neuroinflammatory signaling and modulation of the gut–brain axis.

*Cydonia oblonga* is a nutrient-rich but underutilized horticultural product with reported antioxidant, anti-inflammatory, and immunomodulating properties, suggesting potential benefits in chronic disease prevention [30]. Consistent with these properties, our study demonstrated that HECO effectively reduced immobility time in both the FST and TST in LPS-induced mice. This finding is consistent with a previous report demonstrating that both aqueous and ethanolic concentrates of COM fruit exhibit antidepressant effects in experimental animals, as assessed in behavioral tests such as the FST and TST [31]. The observed behavioral improvements may be partially attributed to the high flavonoid content of the COM fruit, which has been reported to exert neuroprotective and antioxidant effects and may support neurogenesis. However, motor function tests (e.g., rotarod) and general status indicators were not assessed, so non-specific effects on OFT results cannot be excluded.

It is well established that systemic administration of LPS induces robust depressive-like behaviors in rodents through activation of the peripheral immune system and subsequent propagation of inflammatory signaling to the brain [32,33]. In the present study, LPS administration significantly increased spleen weight, a classical marker of systemic immune activation characterized immune cell proliferation and inflammatory responses in peripheral lymphoid organs [34,35]. Importantly, HECO pretreatment significantly attenuated LPS-induced splenomegaly, suggesting that its antidepressant-like effects may, at least in part, be associated with modulation of peripheral immune activation.

The systemic inflammatory response induced by LPS is characterized by the robust release of pro-inflammatory cytokines and mediators [36]. Our findings demonstrated that HECO significantly mitigated this response in a targeted manner. Specifically, HECO significantly reduced serum IL-6 and COX-2 levels. IL-6 is a pleiotropic cytokine critically involved in the pathogenesis of depression, facilitating communication between the peripheral immune system and the brain [37]. Similarly, COX-2 is an inducible enzyme that promotes the production of pro-inflammatory prostaglandins at sites of inflammation [38]. Notably, HECO did not significantly alter TNF-α, IL-1β, or iNOS levels, suggesting potential selective modulation of inflammatory mediators rather than a broad suppression of the inflammatory response. This selective regulation may contribute to the attenuation of the overall inflammatory cascade and represents a possible mechanism underlying the anti-inflammatory effects of HECO in LPS-induced systemic inflammation. Moreover, IL-6 and COX-2 have been implicated in the pathogenesis of depression, particularly in LPS-induced neuroinflammation and sickness behavior [39]. Therefore, the preferential modulation of these mediators may contribute to the antidepressant-like effects of HECO without inducing excessive immunosuppression.

Furthermore, we investigated the underlying molecular pathways within the hippocampus, a brain region highly vulnerable to neuroinflammation. HECO treatment markedly reversed the LPS-induced upregulation of TLR4, MyD88, and NF-κB, suggesting modulation of the TLR4/MyD88/NF-κB signaling pathway. As NF-κB is a master transcription factor controlling the expression of various inflammatory genes, its inhibition provides a plausible explanation for the observed reduction in inflammatory mediators [40]. In parallel, LPS exposure reduced the expression of the tight junction proteins Occludin and ZO-1 in the hippocampus, whereas HECO restored their levels. These proteins are essential components of endothelial tight junctions and are widely used as indicators of BBB integrity [41]. Disruption of BBB integrity has been associated with neuroinflammatory processes and depression-like phenotypes [42]. Taken together, these findings suggest that HECO may alleviate LPS-induced neuroinflammation, potentially through modulation of the TLR4/MyD88/NF-κB signaling pathway and preservation of hippocampal tight junction proteins. These changes may be associated with the attenuation of neuroinflammatory burden and improvement of depressive-like behaviors.

The gut microbiota plays a pivotal role in modulating the gut–brain axis, and its dysbiosis is increasingly implicated in the pathophysiology of depression [9,43]. Our 16S rRNA sequencing data revealed that HECO administration altered the gut microbial community structure in LPS-induced mice, as indicated by the distinct separation among groups in the PCoA plot. HECO treatment resulted in a decrease in the Chao index, suggesting changes in microbial richness. Although reduced richness is often associated with dysbiosis, it may also reflect ecological restructuring of the microbial community. Therefore, the functional implications of this observation remain to be fully elucidated. While the ratio of the two dominant phyla, Bacteroidota and Firmicutes (B/F ratio), remained unchanged, we observed a significant and specific increase in the phylum Deferribacterota following HECO treatment. This phylum, particularly its representative genus Mucispirillum, has been associated with mucosal integrity and anti-inflammatory effects in the gut [44]. Whether these microbial changes directly contribute to the antidepressant-like effects of HECO remains to be determined. Further studies (e.g., fecal microbiota transplantation or gnotobiotic models) are needed to establish a causal link.

Further analysis at the genus level identified Parabacteroides as a differentially abundant genus enriched in the HECO group. This finding is noteworthy, as Parabacteroides has been reported to exhibit anti-inflammatory properties and to produce beneficial metabolites like succinate [45]. Interestingly, reduced abundance of Parabacteroides has been observed in chronic restraint stress-induced depression models, and its levels have been negatively correlated with depressive-like behaviors [46]. Conversely, the LPS group was characterized by an enrichment of Parasutterella, a genus that has been associated with irritable bowel syndrome and intestinal chronic inflammation [47]. Notably, the observed microbial alterations suggest that HECO may promote a gut microbial profile associated with reduced inflammatory potential, which could contribute to the modulation of systemic immune responses. Importantly, these microbiota changes may act upstream of systemic inflammation and neuroinflammatory signaling, potentially complementing the inhibition of the TLR4/MyD88/NF-κB pathway observed in the hippocampus.

Consistent with these findings, serum metabolomic analysis revealed substantial metabolic remodeling following HECO treatment. A total of 262 differential metabolites were identified between the LPS and HECO groups, with the top potential biomarkers predominantly categorized into lipids, flavonoid-containing phenylpropanoids, organic acids, and peptides. Lipids, especially glycerophospholipids, emerged as the most prominently regulated class and are associated with inflammatory responses and membrane homeostasis, key processes perturbed in LPS-induced depression [48,49,50]. Given that flavonoids such as rutin and catechin are major bioactive components of HECO and have been reported to influence lipid metabolism, the observed metabolic changes may be partly related to the regulation of lipid-associated inflammatory pathways [51]. Collectively, these metabolomic alterations suggest that HECO may contribute to the improvement of LPS-induced depressive-like behaviors through coordinated regulation of lipid metabolism and inflammatory signaling, although causal relationships require further validation.

**Figure 10:**
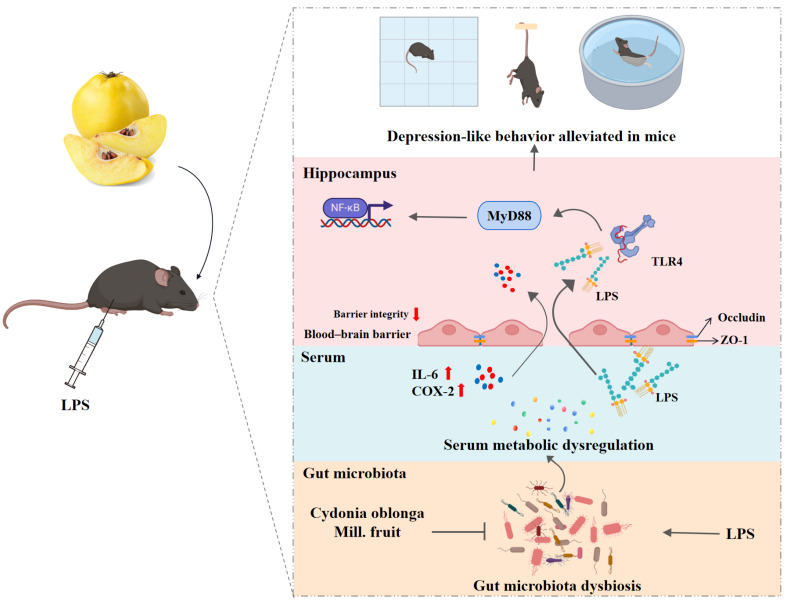
The proposed mechanistic model of HECO against LPS-induced depression-like behavior in mice. HECO administration modulates gut microbiota composition and serum metabolites, selectively reduces systemic inflammation (IL-6 and COX-2), inhibits hippocampal TLR4/MyD88/NF-κB signaling, and restores tight junction proteins (occludin and ZO-1), collectively leading to the improvement in depressive-like behaviors. Solid black arrows represent promoting/activating biological effects; black T-shaped lines indicate inhibitory effects exerted by HECO; upward red arrows mark elevated levels of IL-6 and COX-2; the downward red arrow represents reduced blood-brain barrier integrity; multicolored scattered dots represent differential serum metabolites; distinct colored bacterial shapes stand for different gut microbial strains; pink, light blue and beige background zones separately label hippocampus, serum and gut microbiota compartments.

KEGG enrichment analysis further suggested that HECO-related metabolic alterations were mainly associated with glycerophospholipid metabolism, PI3K-Akt signaling, mTOR signaling, and retrograde endocannabinoid signaling pathways. These pathways may interact with the TLR4/MyD88/NF-κB axis, forming a regulatory network involved in neuroinflammation and synaptic plasticity. Multi-omics analyses have highlighted the important role of glycerophospholipid metabolism in the crosstalk between gut and brain in depression [52]. The PI3K-AKT/mTOR pathway is a critical target for mediating the rapid antidepressant effects of pharmacological agents in clinical and preclinical research [53]. In addition, the retrograde endocannabinoid pathway has been implicated in the pathophysiology of major depressive disorder at both molecular and genetic levels [54]. Collectively, these metabolomic findings indicate HECO may exert antidepressant effects through the integration of gut microbiota modulation, inflammatory suppression, and metabolic homeostasis. Flavonoids may contribute to the crosstalk between metabolic cascades and inflammatory signaling, although this requires further experimental validation. Previous cellular studies have demonstrated that *Cydonia oblonga* flavonoids can suppress NF-κB activation and reduce oxidative stress in vitro [19,55], supporting our in vivo observations. However, the present study did not include in vitro experiments to directly verify these cellular mechanisms. Based on the above findings, we propose a mechanistic model summarizing the potential pathways by which HECO alleviates LPS-induced depressive-like behavior, as illustrated in Figure 10.

Despite these encouraging findings, several limitations should be acknowledged. First, a HECO-treated healthy control group was not included, which limits the assessment of its direct effects in healthy animals, although no obvious toxicity was observed. Second, the relatively small sample size (*n* = 6 per group) may reduce statistical robustness, particularly in multi-omics analyses, and independent validation cohorts are required to confirm the findings. Third, due to the lack of functional intervention experiments (e.g., pathway inhibition, microbiota depletion, fecal microbiota transplantation, or metabolite rescue), the observed associations cannot be interpreted as causal relationships. Fourth, the stability, pharmacokinetics, and storage conditions of HECO were not systematically evaluated, and the specific roles of individual flavonoid components warrant further investigation. Finally, this study did not include in vitro mechanistic experiments using relevant cellular models, which limits the resolution of cell type-specific effects. Future studies incorporating these approaches will be necessary to further elucidate the mechanisms underlying the antidepressant-like effects of HECO.

## 5. Conclusions

In conclusion, HECO, a flavonoid-rich extract from *Cydonia oblonga*, exerts antidepressant-like effects in LPS-induced mice. These effects are associated with the modulation of systemic inflammation, as evidenced by the selective reduction in IL-6 and COX-2, and the regulation of the TLR4/MyD88/NF-κB signaling pathway in the hippocampus. In addition, HECO administration was accompanied by alterations in gut microbiota composition and changes in serum metabolic profiles, particularly in glycerophospholipid metabolism. These multi-level biological changes may collectively contribute to the observed behavioral improvements. Taken together, our findings suggest that HECO may represent a promising natural candidate for the management of inflammation-related depression and highlight the potential value of *Cydonia oblonga*-derived flavonoids in preclinical mental health research.

## Data Availability

All datasets generated in this study are available from the corresponding author on reasonable request.

## Abbreviations

The following abbreviations are used in this manuscript:

HECO	hydroethanolic extract of *Cydonia oblonga* Mill. fruit
LPS	lipopolysaccharide
WHO	World Health Organization’s
TLR4	toll-like receptor 4
MyD88	myeloid differentiation factor 88
NF-κB	nuclear factor-κB
IL-6	interleukin-6
TNF-α	tumor necrosis factor-α
COX-2	cyclooxygenase-2
iNOS	inducible nitric oxide synthase
OFT	Open-field test
TST	Tail suspension test
FST	Forced swim test
ZO-1	zonula occludens-1
